# Knowledge, attitude and practice of emergency contraceptive pills among community pharmacy practitioners working in Kathmandu Valley: a cross-sectional study

**DOI:** 10.1186/s12913-020-05543-5

**Published:** 2020-07-29

**Authors:** Sujyoti Shakya, Sweta Shrestha, Rojeena Koju Shrestha, Usha Giri, Sunil Shrestha

**Affiliations:** 1 Department of Pharmaceutical Science, Nobel College, Affiliated to Pokhara University, Sinamangal, Kathmandu, Nepal; 2 Department of Pharmaceutical and Health Service Research, Nepal Health Research and Innovation Foundation, Lalitpur, Nepal; 3 Department of Pharmacy, School of Sciences, Kathmandu University, Dhulikhel, Kavre Nepal; 4 Department of Pharmacy, Nepal Cancer Hospital and Research Center, Harisidhhi, Lalitpur, Nepal; 5 Department of Nursing, Nobel College, Affiliated to Pokhara University, Sinamangal, Kathmandu, Nepal

**Keywords:** Community pharmacy, Emergency contraceptive pills, Emergency contraception, Nepal, Pharmacist

## Abstract

**Background:**

Unintended pregnancy occurs due to incorrect or inconsistent use of a contraception method. Such pregnancies can create an economic burden on the family, society and nation as a whole. Unintended pregnancy is the underlying cause of abortion which can also result in infertility and maternal death. Adequate knowledge of emergency contraceptive pills (ECPs) and positive attitudes among the community pharmacy practitioners (CPPs) is a prerequisite for timely access of ECP, thus ultimately lessening the incidence of unintended pregnancies. This study intended to explore the knowledge, attitude and practice of CPPs toward ECPs in Kathmandu valley.

**Methods:**

Cross-sectional study conducted in community pharmacies located in three districts of Kathmandu valley. A convenience sampling method was employed to interview CPPs in 227 community pharmacies using a validated questionnaire. Questionnaire assessed the demographic characteristics; knowledge, attitude and dispensing practice of the CPPs. Data were subjected to descriptive and inferential analysis using SPSS 18 (SPSS Inc., Chicago, IL, USA).

**Results:**

Approximately 75% of respondents had a good practice on dispensing ECPs, and 70% of them counselled all the users. A significant association (*p*-value< 0.05) was obtained between the dispensing practice of respondents and their knowledge level. ECP related knowledge was higher among the age group 40–49 years, BPharm degree holders with experience above 10 years and community pharmacies located inside the city and in the Kathmandu district. After adjusting the possible confounder variables, age, degree and district of pharmacy were significantly associated with knowledge. Similarly, respondents’ practice towards ECP was higher among the age group 40–49 years with experience above 10 years and community pharmacies located inside the city and in the Kathmandu district. Adjusted for other variables, only community pharmacies located at Kathmandu district was significantly associated with the practice.

**Conclusion:**

CPPs lacked specific important information on ECP and opined against its’ availability as an over-the-counter drug, despite good overall knowledge and positive attitude. Many thought that ECP without prescription would increase promiscuity towards sexual behaviour and result in unsafe sex along with its’ repeated use. Hence, training and proper counselling strategies should be afoot to refine the delivery of service by CPPs.

## Background

Unintended pregnancy is a pregnancy that usually includes either unwanted or mistimed pregnancy which occurs due to failure to choose an effective contraception method or its’ incorrect and inconsistent use [1, 2]. Such pregnancies can place an economic burden on the nation as a whole and can put the women at reproductive health risk. Abortion can be a possible sequel which further can lead to infertility and maternal death [3, 4]. Globally, it is estimated that 22% of such unintended pregnancies are terminated with unsafe techniques, and 18% end up in unplanned births thus imposing an economic burden on the health system [2, 5]. According to the Nepal Demographic and Health Survey (NDHS) 2011, more than one in five births (21%) is unwanted and one in seven (14%) is mistimed [6] and 58% of women succumb to complications of clandestine abortion [7]. Early age at the time of menarche and late marriage are the underlying factors contributing to pre-marital sex and unintended pregnancies [8]. Ten to 20 % of adolescents in Nepal participate in pre-marital sex. Out of them, only 9% utilize a technique for contraception [9].

Emergency contraception (EC) is any method of contraception that women can use after unprotected sexual intercourse and before the potential time of implantation [10]. Emergency contraceptive pills (ECPs) are safe and effective drugs used to prevent the risk of pregnancy after unprotected or inadequately protected sexual intercourse [11]. ECP was first introduced in Nepal through the social marketing program held in 2004 [12]. Nevertheless, various barriers exist that keep young women from having easy access to ECPs. These barriers incorporate healthcare provider’s knowledge and perception, distribution system for ECPs, legal and social barriers and cost [13].

Community pharmacies stand as an essential access point for obtaining ECPs as well as serve as a salient venue offering counselling services to the public and the first place to approach for drugs owing to its’ flexible time of operation [14–16]. In a woman's access to ECPs, community pharmacies play a pivotal role, which is further influenced by the community pharmacy practitioners (CPPs) knowledge and attitude [14, 15]. Appropriate knowledge and a positive attitude are crucial elements that enable the CPPs to provide comprehensive counselling and create awareness regarding ECP and maximize its rational use, thus preventing unwanted risk [17, 18]. CPPs denote not only pharmacists who have a degree of Bachelor of Pharmacy, Diploma of Pharmacy, or Masters of Pharmacy but also other groups of individuals who have obtained a license from a short orientation training course before the development of pharmacy education in Nepal. Apart from them, Community Medicine Assistant (CMA) and health assistant also dispense medication; however, they are not considered as CPPs. In Nepal, three levels of pharmacy workers dispense medication which includes: Pharmacists who have acquired a bachelor’s degree in pharmacy (BPharm) after twelve years of schooling; Assistant Pharmacists who have completed Diploma in Pharmacy (DPharm) course after ten years of school; and “professionals” who only completed a short orientation training program. However, only pharmacists and assistant pharmacists can register with the Nepal Pharmacy Council [16, 19] and become eligible to run community pharmacy. In 1980, the 48-h orientation training course was introduced and at that point offered to people involved in the pharmaceutical business [20]. Over time, the span of the orientation course was stretched out to 72 h. Regardless of development in this period, the training course was still found to be deficient. It was recommended to discontinue the short orientation course and support the individuals linked with the diploma course in pharmacy [21]. This training has not been led throughout the previous twelve years. All three categories have equal rights to provide pharmaceutical services and to run community pharmacies. In context of Nepal, most of the registered pharmacies in Nepal are run by “professionals” [20–22]. Certain paramedical personnel, typically health assistants (HAs), and community medicine assistants (CMAs) also manage community pharmacies in Nepal. HAs and CMA have completed basic medical training for 36 months and 18 months, respectively, who have completed 10 years of schooling [21]. However, HAs and CMA are not allowed to register a community pharmacy in Department of Drug Adminstartion (DDA).

To the best of our knowledge and search, no previous published researches are investigating the knowledge, attitude and practice (KAP) of CPPs of Nepal on ECP However, the level of knowledge, attitude and practices of emergency contraceptives among college students and other female users have been studied [23, 24]. With this background, this study was thus initiated considering the lack of information regarding KAP of CPPs towards ECPs in Kathmandu Valley.

## Methods

### Study design and study site

A cross-sectional study carried out at community pharmacies located in Kathmandu valley from May 2019 to October 2019. Kathmandu valley consists of 3 districts; Kathmandu, Lalitpur, and Bhaktapur, of Province Bagmati of which the total population is 2,472,071 and the total area are 902.61 km^2^ (348.50 sq. mi). Kathmandu is the capital city, and the three districts are located in the central part of Nepal. The current study was restricted to these areas because urban agglomeration of these 3 districts covers a large population due to the facilities such as employment opportunities, health and transportation facilities and so on. Besides that, these three districts lie in the Central Development Region and Province Bagmati of Nepal. In 2014, the unintended pregnancy rate for Nepal was 68 per 1000 women of reproductive age [25] and ranged from 47 per 1000 women in the Far-Western region to 85 per 1000 in the Central development region [25].

Moreover, abortion rates in Nepal in 2014 ranged from 21 per 1000 reproductive-age women in the Far-Western development region to 59 per 1000 in the more urban Central development region, which includes the capital city of Kathmandu, Lalitpur and Bhaktapur [25]. Several factors may describe the reason for the higher unintended pregnancy rate and abortion rate in the Central region of Nepal [25]. In comparison to other developing regions, the couples those in or near the capital are intended to have smaller families due to various reasons such as higher expenses, busy life, and so forth. Due to the easy accessibility of abortion procedures in urban areas, there is the likelihood of pre-marital sex which results in unintended pregnancy and abortion [25]. Hence, to limit such incidences, there should be knowledge and awareness about ECPs.

### Study population

The study population included the community pharmacies listed in the Department of Drug Administration (DDA) directory. According to the DDA directory, the total registered allopathic community pharmacy of whole Nepal was 12,865 and the total registered allopathic community pharmacy in the three districts of Kathmandu Valley was 2871 till November 2018.

### Inclusion criteria

- Community pharmacies located at three districts of Kathmandu valley, i.e. Kathmandu, Lalitpur and Bhaktapur.
- Community pharmacies registered in the Department of Drug Administration (DDA).
- CPPs working at community pharmacies willing to participate in the study.

### Exclusion criteria

- CPPs not willing to participate in the study and community pharmacies not registered in DDA.
- Pharmacies located inside the hospital and the hospital pharmacies.

### Sampling method and technique

The sample size was calculated using the list of registered community pharmacies obtained from the DDA directory. The community pharmacies located at Kathmandu, Lalitpur and Bhaktapur districts were used for the sampling frame. The Raosoft sample size calculator was used for calculating the required sample size (i.e. 227 calculated) with a 5% margin of error, 95% confidence interval, and 20% response distribution [26]. Questionnaire was distributed to a random sample of CPPs of community pharmacies by using a convenience sampling method. After that, the sample was stratified by three districts of Kathmandu valley. As per the stratification result, the samples taken from Kathmandu, Lalitpur and Bhaktapur districts were 170, 37 and 20 respectively.

### Variables

#### Independent variables

The independent variables included characteristics related to socio-demography and work profile of CPPs. Socio-demographic variables included age, gender, religion and educational status. The work profile included the primary position of CPPs at working place, years of experience, location of the pharmacy, and district of pharmacy.

#### Outcome variables

The dependent variables included knowledge, attitude and practice (KAP) of CPPs working in the community pharmacy on ECPs. Community pharmacies play a pivotal role in a woman’s access to ECPs and the CPPs need to have sufficient knowledge and a positive attitude towards the use of contraceptives to maximize its rational use. Access, use and availability of ECPs are, however, influenced by pharmacy personnel’s knowledge and attitudes towards them. Studies have reported that a lack of knowledge and negative attitudes among the pharmacists hinder women’s timely access to ECPs [14, 15, 17, 18].

Hence, we chose KAP as the dependent variables, and the independent variables were chosen based on previous similar studies done in other countries to find out the association between the independent and the dependent variables. This study will generate the baseline data on KAP of community pharmacists on ECP, which will further help in developing future interventional studies that will ultimately aid in promoting rational use of ECP.

### Data collection tool

A self-administered questionnaire (Additional file 1) was designed after reviewing the previous similar surveys with some modifications [27]. After development of the data collection questionnaire , it was subjected to a review and a validation process. Developed questionnaire was then verified for readability and ease of understanding among 23 CPPs (10% of 227), which were randomly selected CPPs working in different community pharmacies with similar settings to the study site. Face validation of the questionnaire was carried out by colleagues from the pharmacy department and content validation was finalized by discussing the questionnaire to content experts of community pharmacy, pharmacy practice, public health, consumers and statistician within the country. Internal consistency of the questionnaire was measured by calculating Cronbach’s alpha value, which was found to be 0.70, which means there are acceptable reliability and consistency between the set of test items. An alpha of 0.70 indicates acceptable reliability and 0.80 or higher indicates good reliability [28]. Except for the adjustment of the questionnaire such as rephrasing sentence, grammar, wording and language, the outcomes of the pre-testing was excluded in the final data analysis.

The final version of the data collection questionnaire included four sections. Section 1 comprised of 8 items questionnaire that explored demographic and related information of CPPs: age, gender, religion, education(degree), primary position at workplace, years of working experience, location of pharmacy, and district in which pharmacy is situated. Section 2 of the questionnaire comprised of 14 items, which was designed to evaluate the practice of CPPs on ECPs. Section 3 comprised of 10 items designed to evaluate knowledge of CPPs on ECPs and section 4 included 13 items designed to evaluate attitude of CPPs on ECPs.

The questionnaire consisted of both closed-ended and open-ended questions. The questionnaire was hand-delivered and was framed in the English language.

### Process of data collection

First of all, CPPs were approached by the pharmacist from the study team, and the objectives of the study were explained. Written informed consent from the respondents was taken and was assured that their participation in this study was voluntary, and their confidentiality would be maintained. The final data collection tool (obtained after the pilot study) was distributed to CPPs working at different community pharmacies at Kathmandu valley. Thirty to forty-five minutes given to respondents for completing and returning the questionnaire required to CPPs.

### Scoring system

To assess the dispensing practice and knowledge of the respondents on ECP, selected variables were used and, then the correct answer was coded as ‘yes’ which means ‘1’ and an incorrect answer was coded as ‘No’ which means ‘0’. The cumulative and mean scores were calculated. Respondents who scored above the mean score were defined as having “good practice” and good knowledge, and those who scored below the mean score were defined as having “poor practice” and “poor knowledge”. The attitude of respondents was calculated with the help of a five-point Likert scale ranging from; ‘Strongly agree,’ i.e. ‘1’ to ‘Strongly disagree’ i.e. ‘5’. Based on the cumulative score, the respondents who scored below the mean score were defined as having a “positive attitude”, and those who scored above the mean score were defined as having a “negative attitude”.

### Data management and analysis

Data processing and analysis were done by using statistical package for social sciences (SPSS) version 18 (SPSS Inc., Chicago, IL, USA). Data were analyzed for descriptive and inferential statistics. Descriptive analysis was performed using frequencies and percentages and was presented in the form of text, figures, and tables. The Pearson Chi-square test (*X*^*2*^) was used to determine associations among categorical variables. A *p*-value < 0.05 was considered as statistically significant. Crude, as well as Adjusted odds ratios and 95% confidence intervals, was derived from bivariate and multivariate logistic regression models respectively to identify determinant variables associated with dispensing practices, knowledge, and attitudes; crude and adjusted odds ratios were considered significant at *p* ≤ 0.05.

## Results

### Socio-demographic characteristics

Table 1 shows the socio-demographic information of the CPPs working at the community pharmacies. A total of 227 CPPs participated in the study. Majority of the respondents were of the age group 20–29 (47.1%). Among the total respondents, 57.7% were male, and most of them had a Diploma in Pharmacy (DPharm) degree. The highest number of respondents had a work experience of fewer than 5 years and a median length of work experience in the current study was found to be 5–10 years.

**Table 1:** Socio demographic characteristics of the respondents

Characteristics	Frequency	Percentage
**Age** (Median Age: 20–29 Years)		
< 20	29	12.8
20–29	107	47.1
30–39	51	22.5
40–49	23	10.1
Greater and equal to 50	17	7.5
**Gender**		
Male	131	57.7
Female	96	42.3
**Religion**		
Hindu	190	83.7
Buddhist	32	14.1
Muslim	3	1.3
Christian	2	.9
**Degree/Education**		
Bachelor of Pharmacy	57	25.1
CMA	29	12.8
Diploma in Pharmacy	90	39.6
Masters of Pharmacy	13	5.7
Others	38	16.7
**Primary Position**		
Staff	109	48.0
Manager	12	5.3
Owner	105	46.3
Others	1	.4
**Years of Experience** (Median: 5–10 Years)		
< 5 years	111	48.9
5-10 years	61	26.9
> 10 years	55	24.2
**Location of Pharmacy**		
Inside city	123	54.2
Near Hospital	69	30.4
Periphery	35	15.4
**District in which pharmacy is situated**		
Kathmandu	170	74.9
Lalitpur	36	15.9
Bhaktapur	21	9.3

### Dispensing practice of ECPs

Table 2 outlines the dispensing practice of CPPs. The overall dispensing practice of ECP in community pharmacies was found to be good. Out of 227 CPPs, 99.1% reported that they had dispensed ECP. Majority (67%) of CPPs stated that on average they dispensed 1–10 range of ECPs daily.

**Table 2:** Percentage distribution of respondents by their dispensing practice of ECP

S.N	Practice Variables	Response	Percentage distribution of respondents *n* (%)
1	Have you ever dispensed ECP?	Yes	225 (99.1)
		No	2 (0.9)
2	Which brand of ECP is sold the most?	I Pill	129 (56.8)
		E-72	25 (11.0)
		ECON	28 (12.3)
		Unwanted 72	35 (15.4)
		Max 72	10 (4.4)
		Feminor	0
		Others	0
3	On average, how many ECPs do you dispense every day?	1 to 10	151 (66.5)
		11 to 20	63 (27.8)
		21 to 30	11 (4.8)
		31 to 40	1 (0.4)
		41 to 50	1 (0.4)
		Above	0
4	Who are the most frequent clients?	Teenagers	132 (58.1)
		Adult women	49 (21.6)
		Adult men	46 (20.3)
5	Most often the products are sold on?	Patient Request	204 (89.9)
		Patient approaches with prescription	16 (7.0)
		On your recommendations	7 (3.1)
6	Source of information	Medical Representative (MR)	66 (29.1)
		Text Book	99 (43.6)
		Training	44 (19.4)
		Internet	18 (7.9)
7	Do you feel ECPs should be categorized under OTC drug?	Yes	70 (30.8)
		No	123 (54.2)
		Don’t Know	34 (15.0)
8	Received formal training /education on dispensing of ECP	> 1 year back	56 (24.7)
		< 1 year back	24 (10.6)
		Not received	147 (64.8)
9	Do you think counseling is an important role of dispensers before dispensing ECPs	Yes	209 (92.1)
		No	9 (4.0)
		Don’t Know	9 (4.0)
10	Do you counsel all ECP users while dispensing?	Yes	159 (70.0)
		No	68 (30.0)
11	Do you counsel on the mechanism of action of ECPs?	Yes	105 (46.3)
		No	122 (53.7)
12	Do you counsel the time at which ECPs should be taken?	Yes	167 (73.6)
		No	60 (26.4)
13	Do you counsel on the side effects of ECPs?	Yes	151 (66.5)
		No	76 (33.5)
**Summary of Selected Practice Variables**			
**(Mean Score)**		**Number**	**%**
Poor Practice (≤0.5)		57	25.1
Good Practice (> 0.5)		170	74.9
Total		227	100.0

### Association of demographic characteristics and dispensing practice

Chi-square test was used to determine the association between socio-demographic characteristics and dispensing practice, as presented in Table 3. There was a significant association of age, primary position, years of experience, location of community pharmacy and district in which the community pharmacy is situated with dispensing practice (*p* < 0.05) whereas gender, religion, and level of education were not significantly associated with dispensing practice (*p* > 0.05).

**Table 3:** Demographic characteristics of the respondents and their dispensing practice

Variables	Selected Practice Variables		Chi-Square value	*p*-value
	Poor Practice ***n*** (%)	Good Practice ***n*** (%)		
**Age (years)**				
< 20	18 (31.6)	11(6.5)	30.502	0.000*
20–29	27(47.4)	80(47.1)		
30–39	10(17.5)	41(24.1)		
40–49	1(1.8)	22(12.9)		
Greater and equal to 50	1(1.8)	16(9.4)		
**Gender**				
Male	31(54.4)	100(58.8)	0.344	0.557
Female	26(45.6)	70(41.2)		
Religion				
Hindu	45(78.9)	145(85.3)	3.774	0.287
Buddhist	9(15.8)	23(13.5)		
Muslim	2(3.5)	1(0.6)		
Christian	1(1.8)	1(0.6)		
**Degree**				
B Pharmacy	11(19.3)	46(27.1)	7.004	0.136
CMA	12(21.1)	17(10.0)		
D Pharmacy	20(35.1)	70(41.2)		
M Pharmacy	2(3.5)	11(6.5)		
Others	12(21.1)	26(15.3)		
**Primary Position**				
Staff	39(68.4)	70(41.2)	13.476	0.004*
Manager	3(5.3)	9(5.3)		
Owner	15(26.3)	90(52.9)		
Others	0(0.0)	1(0.6)		
**Years of Experience**				
< 5 years	35(61.4)	76(44.7)	12.367	0.002*
5–10 years	18(31.6)	43(25.3)		
> 10 years	4(7.0)	51(30.0)		
**Location of Pharmacy**				
Inside city	24(42.1)	99(58.2)	7.947	0.019*
Near Hospital	18(31.6)	51(30.0)		
Periphery	15(26.3)	20(11.8)		
**District in which pharmacy is situated**				
Kathmandu	17(29.8)	153(90.0)	82.226	0.000*
Lalitpur	25(43.9)	11(6.5)		
Bhaktapur	15(26.3)	6(3.5)		

* indicates statistically significant at *p*-value less than 0.05

### CPPs knowledge on ECPs

Table 4 depicts the knowledge of respondents towards ECP. Approximately 149 (65.6%) had good knowledge, while 78 (34.4%) had poor knowledge of ECPs. The fact that ECPs work by preventing or delaying ovulation was agreed upon by the majority of the respondents (63.4%). When asked about how many times in the past years they had received information about ECP, only a lower percentage of the CPPs (21.1%) responded: “yes, more than once”. Majority (81.5%) believed that ECPs should be taken after unprotected sexual intercourse to be clinically effective. Quite interestingly, a majority of the respondents presumed that “Levonorgestrel” is the main chemical constituents of ECP (76.7%). When asked about the dose of ECP, the majority (68.3%) of the respondents reported the dose as “Single dose of 1.5 mg or 2 doses of 0.75mg”. Furthermore, 63.4% incorrectly stated that ECP could harm a developing fetus. A majority of the respondents (75.3%) correctly believed that ECP does not offer protection against sexually transmitted infections (STI). Majority of the CPPs (67.4%) responded that they know about the side effects of ECP.

**Table 4:** Percentage distribution of respondents by their knowledge of ECP

Knowledge variables	Response	Percentage n (%)
Mechanism of action of ECP	Prevent or delay ovulation	144 (63.4)
	Induce Abortion	26 (11.5)
	Prevent an already established pregnancy	23 (10.1)
	Don’t Know	34 (15.0)
How many times in the past years have you received information about ECP?	No	83 (36.6)
	Yes, Once	96 (42.3)
	Yes, More than once	48 (21.1)
Do you know when must the pills be taken to be clinically effective?	Before unprotected sexual intercourse	15 (6.6)
	During unprotected sexual intercourse	22 (9.7)
	After unprotected sexual intercourse	185 (81.5)
	Don’t Know	5 (2.2)
Within how many hours after unprotected sexual intercourse should the pills be taken?	5	1 (0.4)
	24	12 (5.3)
	48	20 (8.8)
	72	180 (79.3)
	120	13 (5.7)
	Don’t Know	1 (0.4)
Mention the constituents of ECP	Levonorgestrel	174 (76.7)
	Levonorgestrel plus Ethinyl estradiol	24 (10.6)
	Don’t Know	29 (12.8)
What is the dose of ECPs?	Single-dose of 1.5 mg or 2 doses of 0.75 mg	155 (68.3)
	Single-dose of 2.5 mg or 2 doses of 01.5 mg	22 (9.7)
	Don’t Know	50 (22.0)
ECP can harm the developing fetus?	Yes	144 (63.4)
	No	40 (17.6)
	Don’t Know	43 (18.9)
Do you know the side effects of ECPs?	Yes	153 (67.4)
	No	44 (19.4)
	Not Sure	30 (13.2)
Does the pill protect from Sexually transmitted infections (STI)?	Yes	12 (5.3)
	No	171 (75.3)
	Don’t Know	44 (19.4)
**Summary**		
**Knowledge of ECP (Mean Score)**	**Number**	**%**
Poor Knowledge (≤0.5)	78	34.4
Good Knowledge (> 0.5)	149	65.6
Total	227	100.0

### Common side effects of ECP

**Fig. 1:**
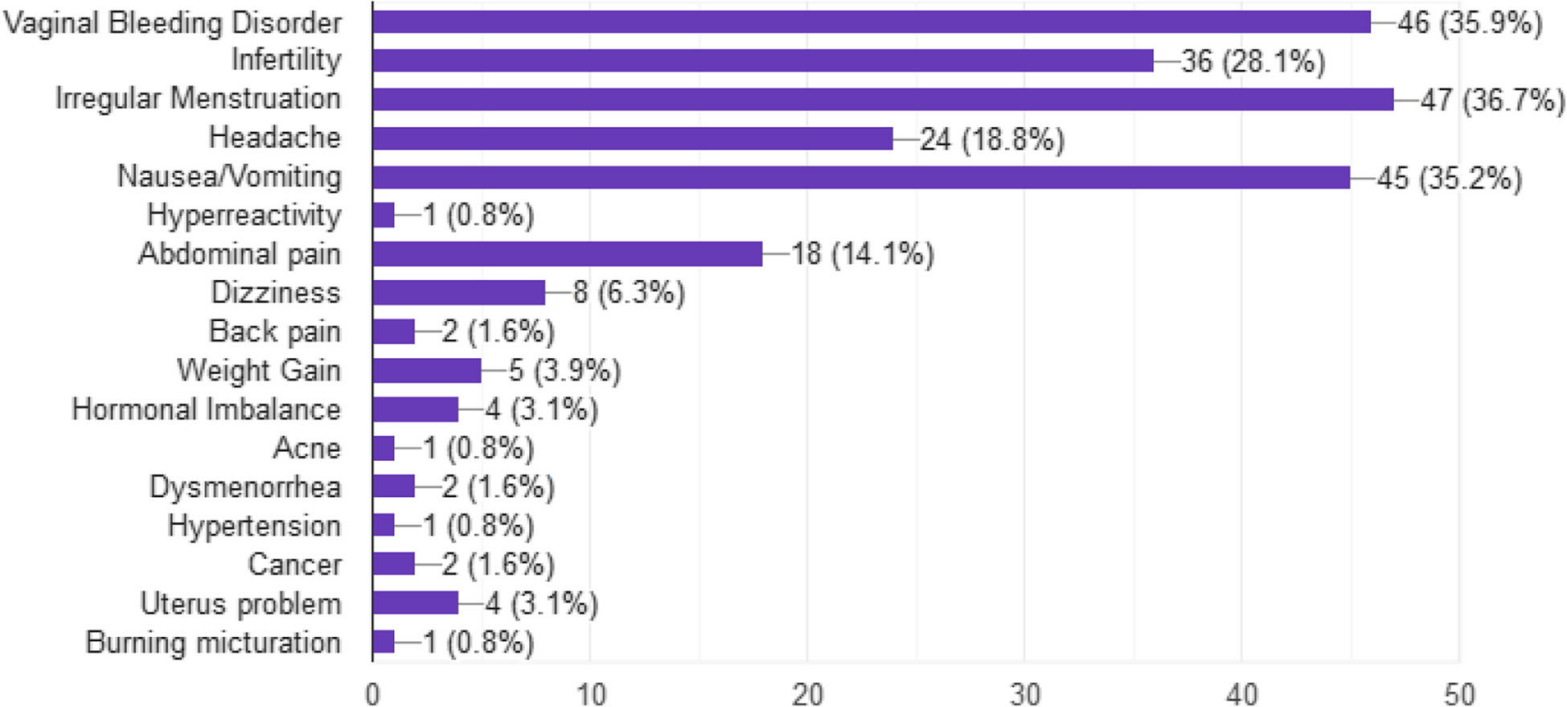
Percentage of common side effects of ECP as specified by respondents

The most common side effects specified by the respondents were irregular menstruation (%) followed by vaginal bleeding (35.9%), nausea/vomiting (35.2%), infertility (28.1%) and headache (18.8%) as presented in Fig. 1.

### Association of demographic characteristics and knowledge level

A significant association was found between age, degree, primary position, years of experience, location of community pharmacy, district of community pharmacy and the respondents’ knowledge level (*p* < 0.05)whereas no significant association was observed between gender, religion and the respondents’ level of knowledge (*p* > 0.05) (Table 5).

**Table 5:** Demographic characteristics of the respondents and their level of knowledge

Variables	Poor knowledge *n* (%)	Good knowledge *n* (%)	chi-Square value	*p*-value
**Age**				
< 20	25 (32.1)	4(2.7)	47.087	0.000*
20–29	37(47.4)	70(47.0)		
30–39	10(12.8)	41(27.5)		
40–49	2(2.6)	21(14.1)		
Greater and equal to 50	4(5.1)	13(8.7)		
**Gender**				
Male	40(51.3)	91(61.1)	2.011	0.156
Female	38(48.7)	58(38.9)		
**Religion**				
Hindu	61 (78.2)	129 (86.6)	3.284	0.350
Buddhist	14 (17.9)	18 (12.1)		
Muslim	2 (2.6)	1 (0.7)		
Christian	1 (1.3)	1 (0.7)		
**Degree**				
B Pharmacy	8(10.3)	49(32.9)	19.954	0.001*
CMA	17(21.8)	12(8.1)		
D Pharmacy	32(41.0)	58(38.9)		
M Pharmacy	4(5.1)	9(6.0)		
Others	17(21.8)	21(14.1)		
**Primary Position**				
Staff	53(67.9)	56(37.6)	19.191	0.000*
Manager	3(3.8)	9(6.0)		
Owner	22(28.2)	83(55.7)		
Others	0(0.0)	1(0.7)		
**Years of Experience**				
< 5 years	48(61.5)	63(42.3)	9.191	0.010*
5–10 years	19(24.4)	42(28.2)		
> 10 years	11(14.1)	44(29.5)		
**Location of Pharmacy**				
Inside city	32(41.0)	91(61.1)	14.106	0.001*
Near Hospital	25(32.1)	44(29.5)		
Periphery	21(26.9)	14(9.4)		
**District in which pharmacy is situated**				
Kathmandu	32(41.0)	138(92.6)	75.583	0.000*
Lalitpur	26(33.3)	10(6.7)		
Bhaktapur	20(25.6)	1(0.7)		

* indicates statistically significant at *p*-value less than 0.05

### Attitude of CPPs towards ECP

Table 6 shows the attitude of respondents towards ECP. Majority of the respondents had a positive attitude towards ECP (93.4%). More than half of the study respondents believed that ECPs are safe to use (53.4%). Only 25.6% reported that adolescents (teenagers) should be given easy access to ECPs and 34% agreed on the recommendation of ECP use. Regarding the medicalization of ECP, 36.5% of the respondents presumed that the government of all countries should medicalize ECPs. The majority of the respondents approved that all of the sexually active women should be aware of ECP (91.6%).

**Table 6:** Percentage distribution of respondents ‘attitude towards ECP

S.N	Attitude Variables	Percentage distribution of respondents attitude towards ECP				
		Strongly Agree ***n*** (%)	Agree ***n*** (%)	Neutral ***n*** (%)	Disagree ***n*** (%)	Strongly Disagree ***n*** (%)
1	ECPs are safe to use	21 (9.3)	100 (44.1)	57 (25.1)	44 (19.4)	5 (2.2)
2	Adolescents (Teenagers) should be given an easy access to ECPs	0	58 (25.6)	58 (25.6)	103 (45.4)	8 (3.5)
3	Do you recommend ECPs use?	4 (1.8)	73 (32.2)	73 (32.2)	64 (28.2)	13 (5.7)
4	Government of all countries should legalize ECPs	16 (7.0)	67 (29.5)	76 (33.5)	63 (27.8)	5 (2.2)
5	All sexually active women should be aware of ECP	64 (28.2)	144 (63.4)	10 (4.4)	9 (4.0)	0
6	Routine information about ECP should be included in contraceptive counseling	64 (28.2)	135 (59.5)	22 (9.7)	6 (2.6)	0
7	Information of ECP should be included in sex education in school	79 (34.8)	135 (59.5)	10 (4.4)	2 (0.9)	1 (0.4)
8	Formal training is needed to enable the dispensers to appropriately dispense ECPs	54 (23.8)	142 (62.6)	22 (9.7)	8 (3.5)	1 (0.4)
9	ECP without prescription will promote unsafe sex	32 (14.1)	116 (51.1)	38 (16.7)	37 (16.3)	4 (1.8)
**Summary**						
**Attitude towards ECP (Mean Score)**		**Number**		**%**		
Negative Attitude (> 3)		15		6.6		
Positive Attitude (≤3)		212		93.4		
Total		227		100.0		

Similarly, a large percentage believed that routine information about ECP should be included in contraceptive counselling (87.7%). Regarding information and formal training of ECP, a maximal percentage favoured that information regarding ECP should also be included in sex education in school (94.3%). In comparison, 86.4% of respondents believed that formal training is needed to enable the CPPs to dispense ECP appropriately. Also, the majority of the respondents believed that ECP without prescription would promote unsafe sex (65.2%).

### Association of demographic characteristics and attitude level of the respondents

No significant association was found between the demographic variables and their level of attitude (*p* < 0.05) (Table 7).

**Table 7:** Demographic characteristics of the respondents and their level of attitude

Variables	Negative Attitude n (%)	Positive Attitude n (%)	Chi-Square value	*p*-value
**Age**				
< 20	3 (20.0)	26 (12.3)	4.370	0.358
20–29	9 (60.0)	98 (46.2)		
30–39	1 (6.7)	50 (23.6)		
40–49	2 (13.3)	21 (9.9)		
Greater and equal to 50	0	17 (8.0)		
**Gender**				
Male	8 (53.3)	123 (58.0)	0.126	0.723
Female	7 (46.7)	89 (42.0)		
**Religion**				
Hindu	12 (80.0)	178 (84.0)	0.777	0.855
Buddhist	3 (20.0)	29 (13.7)		
Muslim	0	3 (1.4)		
Christian	0	2 (0.9)		
**Degree**				
B Pharmacy	4 (26.7)	53 (25.0)	3.601	0.463
CMA	4 (26.7)	25 (11.8)		
D Pharmacy	5 (33.3)	85 (40.1)		
M Pharmacy	1 (6.7)	12 (5.7)		
Others	1 (6.7)	37 (17.5)		
**Primary Position**				
Staff	6 (40.0)	103 (48.6)	2.254	0.521
Manager	2 (13.3)	10 (4.7)		
Owner	7 (46.7)	98 (46.2)		
Others	0	1 (0.5)		
**Years of Experience**				
< 5 years	10 (66.7)	101 (47.6)	2.106	0.349
5–10 years	3 (20.0)	58 (27.4)		
> 10 years	2 (13.3)	53 (25.0)		
**Location of Pharmacy**				
Inside city	8 (53.3)	115 (54.2)	0.093	0.955
Near Hospital	5 (33.3)	64 (30.2)		
Periphery	2 (13.3)	33 (15.6)		
**District in which pharmacy is situated**				
Kathmandu	9 (60.0)	161 (75.9)	1.948	0.378
Lalitpur	4 (26.7)	32 (15.1)		
Bhaktapur	2 (13.3)	19 (9.0)		

### Association of dispensing practice of respondents with their level of knowledge and attitude

A significant association was obtained between the dispensing practice of respondents and their knowledge level (*p* = 0.000), whereas no significant association was found between the dispensing practice of respondents and their level of attitude (*p* = 0.578). The multivariate analysis illustrated that the respondents having good knowledge were 11.86 times more likely to have good practice compared to those having poor knowledge of ECP [AOR = 11.86, 95% CI (5.821–24.190)] (Table 8).

**Table 8:** Association of the level of dispensing practice with their level of knowledge and level of attitude

Variables		Level of Selected Practice Variables		Chi-Square value	*P*-value	COR (95% CI)	AOR (95% CI)
		Poor Practice	Good Practice				
**Level of Knowledge**	Poor Knowledge	43 (75.4)	35 (20.6)	56.941	0.000*	11.847 (5.833–24.060)	1
	Good Knowledge	14 (24.6)	135 (79.4)				11.866 (5.821–24.190)
**Level of Attitude**	Negative Attitude	5 (8.8)	10 (5.9)	0.578	0.537 Fischer Exact Test		
	Positive Attitude	52 (91.2)	160 (94.1)				

*Abbreviation*: *COR* Crude Odds Ratio, *AOR* Adjusted Odds Ratio and * indicates statistically significant at *p*-value less than 0.05

### Association of the level of attitude of respondents with their level of knowledge

Table 9 shows an association of the level of attitude of respondents with their level of knowledge. No significant association was observed between the level of attitude of the respondents and their knowledge level (*p* = 0.109).

**Table 9:** Level of an attitude of respondents and their level of knowledge

Variables		Level of Attitude		Chi-Square value	*p*-value
		Negative Attitude	Positive Attitude		
**Level of Knowledge**	Poor Knowledge	8 (53.3)	70 (33.0)	2.563	0.109
	Good Knowledge	7 (46.7)	142 (67.0)		

### Determinant factors related to knowledge and practice of ECP

- Determinant factors related to respondents’ Knowledge of ECP

Age, degree of education, years of experience, location of community pharmacy and district of community pharmacy are the variables that met the inclusion criteria in multivariate logistic regression analysis as per the results obtained from the bivariate analysis. After subjecting these variables to multivariate analysis, respondents being aged 20–29, 30–39 and 40–49 years, [AOR = 4.779, 95%CI (1.179–19.372), 14.775 (2.456–88.862) and 27.030 (2.043–357.546)] compared to age less than 20 years was significantly associated with knowledge level on ECP with higher odds.

Respondents having Community Medicine Assistant (CMA), DPharm and other degrees were less likely to be knowledgeable about ECP as compared to respondents with a BPharm degree. [AOR = 0.122, 95% CI (0.027–0.537), 0.261(0.080–0.848), 0.128 (0.026–0.629)]. Similarly, respondents whose community pharmacy is located at Lalitpur and Bhaktapur districts were less likely to know about ECP compared to those whose community pharmacy is situated at Kathmandu district [AOR = 0.079, 95% CI (0.024–0.260), 0.010 (0.001–0.125)] after adjusting for the confounding variables (Table 10).

b) Determinant factors related to respondents Dispensing Practice of ECP

**Table 10:** Socio demographic characteristics and determinant variables related to the knowledge and practice of ECP

Variables	Practice Odd Ratio (95% CI)				Knowledge Odd Ratio (95% CI)			
	COR (95% CI)	***p***-value	AOR (95% CI)	***p***-value	COR (95% CI)	***p***-value	AOR (95% CI)	***p***-value
**Age**								
< 20	1	0.000*	1	0.514	1	0.000*	1	0.044*
20–29	4.848 (2.036–11.547)	0.000*	2.538 (0.808–7.970)	0.111	11.824 (3.827–36.536)	0.000*	4.779 (1.179–19.372)	0.028*
30–39	6.709 (2.419–18.606)	0.000*	3.332 (0.761–14.591)	0.110	25.625 (7.256–90.492)	0.000*	14.775 (2.456–88.862)	0.003*
40–49	36.000 (4.236–305.916)	0.001*	5.407 (0.360–81.146)	0.222	65.625 (10.915–394.550)	0.000*	27.030 (2.043–357.546)	0.012*
Greater and equal to 50	26.182 (3.034–225.902)	0.003*	4.142 (0.284–60.418)	0.299	20.313 (4.357–94.697)	0.000*	10.048 (0.971–103.951)	0.053
**Gender**								
Male	1	0.558	–	–	1	0.157	–	–
Female	0.835 (0.456–1.527)		–	–	0.671 (0.386–1.166)	0.157	–	–
**Religion**								
Hindu	1	0.379	–	–	1	0.381	–	–
Buddhist	0.793 (0.342–1.837)	0.589	–	–	0.608 (0.284–1.303)	0.201	–	–
Muslim	0.155 (0.014–1.751)	0.132	–	–	0.236 (0.021–2.658)	0.243	–	–
Christian	0.310 (0.019–5.062)	0.411	–	–	0.473 (0.029–7.687)	0.599	–	–
**Degree**								
B Pharmacy	1	0.15	–	–	1	0.001*	1	0.040*
CMA	0.339 (0.126–0.911)	0.032	–	–	0.115 (0.040–0.330)	0.000*	0.122 (0.027–0.537)	0.005*
D Pharmacy	0.837 (0.367–1.909)	0.672	–	–	0.296 (0.125–0.701)	0.006*	0.261 (0.080–0.848)	0.025*
M Pharmacy	1.315 (0.254–6.807)	0.744	–	–	0.367 (0.091–1.482)	0.159	0.494 (0.079–3.087)	0.451
Others	0.518 (0.201–1.338)	0.174	–	–	0.202 (0.075–0.539)	0.001*	0.128 (0.026–0.629)	0.011*
**Years of Experience**								
< 5 years	1	0.006*	1	0.624	1	0.012*	1	0.375
5–10 years	1.100 (0.557–2.173)	0.783	0.648 (0.245–1.714)	0.382	1.684 (0.871–3.256)	0.121	0.701 (0.234–2.098)	0.525
> 10 years	5.872 (1.967–17.527)	0.002*	1.064 (0.200–5.675)	0.942	3.048 (1.425–6.516)	0.004*	0.292 (0.052–1.641)	0.162
**Location of Pharmacy**								
Inside city	1	0.023*	1	0.931	1	0.001*	1	0.216
Near Hospital	0.687(0.342–1.381)	0.292	0.940 (0.386–2.293)	0.892	0.619 (0.328–1.168)	0.139	0.462 (0.192–1.116)	0.086
Periphery	0.323 (0.145–0.723)	0.006*	1.180 (0.378–3.687)	0.775	0.234 (0.107–0.515)	0.000*	0.590 (0.164–2.116)	0.418
**District of Pharmacy**								
Kathmandu	1	0.000*	1	0.000*	1	0.000*	1	0.000*
Lalitpur	0.049 (0.021–0.116)	0.000*	0.062 (0.024–0.164)	0.000*	0.089 (0.089–0.203)	0.000*	0.079 (0.024–0.260)	0.000*
Bhaktapur	0.044 (0.015–0.130)	0.000*	0.069 (0.021–0.230)	0.000*	0.012 (0.002–0.090)	0.000*	0.010 (0.001–0.125)	0.000*

*Abbreviation*: *COR* Crude Odds Ratio, *AOR* Adjusted Odds Ratio and * indicates statistically significant at *p*-value less than 0.05

Age groups, years of experience, location of community pharmacy and district of community pharmacy were the variables that met the inclusion criteria in multivariate logistic regression analysis as per the results obtained from the bivariate analysis. After adjusting the possible confounder variables in multivariate analysis, respondents whose community pharmacy is situated at Lalitpur and Bhaktapur districts were less likely to have practised on ECP compared to those whose community pharmacy is situated at Kathmandu district. [AOR = 0.062 95% CI (0.024–0.164), 0.069 (0.021–0.230)] respectively as shown in Table 10.

## Discussion

The current study was undertaken to assess the knowledge, attitude, and practice (KAP) of ECP and their associated factors among CPPs of Kathmandu valley. In this study more than half of the respondents were male (57.7%) which was similar to the study conducted in Gondar Town, Northwestern Ethiopia (60%) [29] and Nigeria (57.3%) [18].

### Practice of CPPs on ECP

A vast majority of the respondents in the current study had ever dispensed ECP and the majority of the product was sold on patient requests without a prescription which was similar to the study carried out in Managua, Nicaragua and Ibadan and Lagos Metropolis, Nigeria [18]. Most of the CPPs in our study were willing to dispense ECP to men seeking ECPs for their partner (77.5%) which was similar to the study conducted in Nicaragua (83.9%) [18]. This might be reflective of the fact that men’s participation and support in the use of contraceptive is valued by pharmacy personnel.

Provision of counselling to women seeking emergency contraceptives was reported to be an essential facet of dispensing by 92.1% of respondents, yet only, 70% of the respondents did counsel all the users while dispensing ECP. This statistic was somewhat lower than the studies carried out in Turkey and Ethiopia in which counselling was offered as an essential service by almost all of the pharmacists [29, 30]. This difference may be due to lack of private counselling areas in the community pharmacies of Kathmandu Valley, which was reportedly present in 75% of the pharmacies in Turkey. Furthermore, 53.7, 26.4 and 33.5% did not offer counselling on the mechanism of action of ECP, the timing of ECP intake and its’ side effects respectively which are essential for a woman to get acquainted with, to make choices concerning their reproductive health [31].

Although 86.4% of the respondents agreed to the need of formal training, only 35.3% of the respondents in our study had received any kind of formal training/ education on dispensing of ECP which was consistent with the study conducted in Gondar Town, Northwestern Ethiopia (38.3%) but was lower than the findings by Ehrle et al., and Belachew et al., in which 50% of respondents had received information about the method in the past year [27, 29]. This contrast may be due to the difference in the training facilities in two settings and the lack of awareness of the place and time where the training is conducted. The government and the different pharmacy organizations should take advantage of the enthusiasm of the CPPs and design and run educational campaigns that can aid in mainstreaming ECP use by improving their existing KAP. In the same vein, Kishore et al. pointed out a significant improvement in knowledge, attitude and dispensing practice of the providers after attending training programs on ECP (*p* < 0.05) [32].

In this study, 30.8% of respondents felt that ECPs should be categorized under OTC drugs. This result was slightly lower than the study conducted in Jamaica and Barbados, in which 50.3 and 40.3% of respondents voted for the provision of making it available without a prescription, respectively [33]. This difference may be due to the unsubstantiated belief of the CPPs that ECP without prescription would increase promiscuity towards sexual behaviour and result in unsafe sex along with repeated use of ECP. Therefore, positive aspects of ECPs should be highlighted during training with proper educational messages.

### Knowledge of the respondents towards ECP

This study illustrated that about 149 (65.6%) of the respondents possessed a good knowledge of ECP. The result was slightly lower than the study conducted in New Mexico, in which the pharmacists had overall knowledge scores of 71.2 ± 11.3 [34]. Regarding the mechanism of action of ECP, 63.4% gave the correct answer which was consistent with the study done in Managua, Nicaragua in which more than half of the respondents (59%) knew how the ECPs worked [27]. However, 11.6% of participants believed that ECP could induce abortion. Quite surprisingly, despite this belief of ECP acting as an abortifacient, they were selling it without a prescription which might reflect the financial pressure on the CPPs to earn their livelihood [27]. Majority (81.5%) of the respondents reported that ECP should be taken after unprotected sexual intercourse to be clinically useful which is in agreement with the findings of research conducted on Nicaraguan pharmacists in which this awareness was observed in 79% of the respondents [27, 34]. An unexpected response was obtained from 16.3% of the respondents who recommended the use of ECP before and during intercourse. Most of the respondents reported that ECP should be taken within 72 h after unprotected sexual intercourse, but very few knew that it was also useful if taken within 120 h of unprotected sexual intercourse. This corroborates the findings of studies conducted in Jamaica and Barbados and South Africa [33, 35]. Inadequate knowledge in such important affairs might have significant undesirable effects. For instance, a woman approaching the community pharmacy after 72 h of unprotected intercourse might not receive the legal service and may have to opt for other measures of terminating the resulting pregnancy. A study conducted in South-Eastern Hungary which reported that nearly all (97%) pharmacists were aware of the active agent of the ECP reflects a higher statistic than our study where only 76.7% of the respondents voted for levonorgestrel as the active constituents of ECP. Despite WHO assertion, 57% of respondents in Managua, Nicaragua, and 68% in New Mexico incorrectly believed that ECP could cause harm to the developing fetus versus 63.4% in the current study. Side effects mentioned by the CPPs in this study were no different from those stated by another study [31], except for the mentioning of infertility and cancer as the probable side effects by a few respondents. Such responses might create fear among the women, thus hindering the timely consumption of the ECP and resulting in unwanted pregnancies and the consequent sequel.

In the current study, 67.4% of the respondents knew about the side effects of ECP and 68.3% reported the right dosing, which was lower than the study carried out in South Africa. The reason for this result may be due to the differences in educational levels. Only pharmacists were interviewed in the study conducted in South Africa, whereas all the CPPs irrespective of their degree or level of education were enrolled in the current study. This may be the reason that the present study may have lower knowledge regarding the side effects and dosing schedule of ECP compared to the study conducted in South Africa [35]. A right proportion of the respondents (75.3%) correctly believed that ECP does not offer protection against sexually transmitted infections (STI), underpinning the findings of Szucs et al. [31]. However, 5.3% still believed that ECP is protective against STI. Such a false notion can put the women at significant sexual health risk [31].

A well-informed patient and a well-informed pharmacist are the foundation of a reliable healthcare system. Pharmacists being the information conveyers to the patients thus bear a considerable responsibility to remain adequately prepared and knowledgeable regarding the various contraceptive methods including the ECP to ensure that a woman gets an excellent sexual and reproductive health service. Furthermore, researches conducted among higher secondary students and women of the reproductive age group in Nepal depict the existence of limited knowledge regarding the use of emergency contraceptives [23, 24]. This is indicative of the dire need for educational intervention and training for the CPPs on ECP as they are the first point of contact for customers seeking ECP after unprotected intercourse and thus play an essential role in maintaining good sexual and reproductive health in women [31].

### Attitude of the respondents towards ECP

The current study found that a large majority of the respondents (93.4%) have a positive attitude. More than half of the study respondents agreed that ECPs are safe to use (53.4%), which is similar to the study conducted in Ethiopia [29]. Similarly, a large proportion of respondents agreed that routine information about ECP should be included in contraceptive counselling (87.7%) as well as all sexually active women should be aware of ECP (91.6%). These results were higher in comparison to the study conducted in Ethiopia with a percentage of (75%) and (58.3%) respectively [36] and similar to the study conducted in Turkey with a percentage of (85%) and (92%) respectively [28]. Despite this positive apprehension, a total of 53.4% of respondents disagreed that adolescents should be given easy access to ECPs which was in agreement with research conducted in South Africa in which a significant number of pharmacists doubt their appropriateness for women younger than 18 years of age [33]. Early age marriage between 15 to 19 years is common in Nepal due to illiteracy and poverty that has increased the incidence of adolescent pregnancies which is further escalated by the social pressure of giving birth to a son [37]. Adolescent engagement in pre-marital sex has been reported by several studies done in Nepal, which in many cases has resulted in unwanted pregnancies and subsequent medical or surgical terminations [24, 25]. Amidst such condition, denial of ECP access to minors or imposing age-based restriction by CPPs who are the first point of approach for adolescent females for ECP might surge the occurrence of unintended pregnancies putting them into a reproductive health risk. Regarding the medicalization of ECP, 36.5% of the respondents agreed that the government of all countries should medicalize ECPs while more than two- thirds (68.3%) of the study respondents of Ethiopia and Sweden favoured de-medicalization of ECPs and proposed it to be OTC drug [29, 35]. This difference in result may be due to the concern of CPPs of Kathmandu valley regarding unwise use of ECP by the adolescents and the risk of an increase in unsafe sex, which is evident from the proportion (65.2%) of CPPs consenting to the statement that ECP without prescription will promote unsafe sex. This finding was in line with the study conducted in South Africa in which the majority stated that the use of pills promoted promiscuity, repeat use and increased risk of contracting human immunodeficiency virus (HIV) and other STIs [35], but is contrary to the findings of Apikoglu- Rabus et al. where 52% of the pharmacists believed that teenagers are fully capable of taking responsibility for ECP use [30]. Medicalization of ECP might pose a huge challenge for women seeking emergency contraception due to the obligation of obtaining an appointment from the doctor within the time-frame of 72 h. Hence, the community pharmacy serves as an important facility that offers prompt access to most women seeking ECP within the crucial time-frame, thus safeguarding them from the risk of unintended pregnancies or abortions [30]. This ultimately depends on the CPPs’ attitude and acceptance regarding the use of emergency contraceptives [29].

Majority of the respondents (94.3%) believed that ECP should be a part of comprehensive sexuality education in schools. This data was higher than the study carried out in Turkey, in which only 73.1% of the respondents agreed with the above statement [30]. A course focused on emergency contraceptives and its’ public health benefits can be incorporated in the pharmacy education that could enable pharmacists to offer adequate counseling services to women seeking emergency contraception.

### Determinant factors associated with knowledge and practice of ECP

Age, primary position, years of experience, location of community pharmacy and district of community pharmacy were found to be the determinant factors statistically associated with dispensing practice. Age and years of experience shared a positive relationship with dispensing practice in bivariate analysis and the district in which community pharmacy was situated was found to be a statistically significant factor for dispensing practice in the multivariate analysis. There was no significant association of gender, religion, and level of education with dispensing practice. In the study conducted in Delhi, India, age, years of experience were found to have statistical significance with the dispensing practice of ECP, which was consistent with the current study [32]. Years of experience were found to have a positive relation with dispensing practice in the study conducted in Ethiopia, which was in agreement with the current study [29].

Age, primary position, degree, years of experience, location of community pharmacy and district of community pharmacy was found to be statistically significant factors for the level of knowledge. In bivariate analysis, age and years of experience were found to show positive relation with the level of knowledge. In multivariate analysis, age showed a positive relationship with the level of knowledge of CPPs about ECP whereas years of experience and location in which community pharmacy was situated were found to share negative relation with their level of knowledge. There was no significant association between gender and religion with their level of knowledge. The result is similar to the study conducted in South Dakota state of USA in which years of experience were found to be statistically significant with the level of knowledge [38].

The extent of knowledge of respondents towards ECP was a statistically significant factor for the good dispensing practice of respondents and had a positive relation [AOR = 11.86, 95% CI (5.821–24.190)]. This result was found to be consistent with the studies conducted in India [32] and Florida state of USA [39] in which the dispensing practice of providers were found to be positively correlated with their knowledge (*p* < 0.05).

Even though the majority of the respondents possessed a positive attitude, dispensing practice and knowledge level of the respondents towards ECP did not show any significant association with their level of attitude. However, this result was not consistent with the study done in India [32]. This contradiction may be accounted for the possibility that some of the respondents may have filled in responses they perceived to be desirable rather than their actual perceptions.

However, a positive attitude without adequate knowledge does not correspond with the level of dispensing practice. It is the knowledge that holds a more significant role in decision making in dispensing practice [32].

### Limitations

This study, however, is not without limitations. The study participants were from three districts of Kathmandu valley, i.e. Kathmandu, Bhaktapur and Lalitpur and the findings of this study may not be generalizable to CPPs from other districts of Nepal. Another limitation was the use of convenience sampling method due to which the findings may not be representative of the target population of Nepal. Some of the respondents might have given the answers which the interviewer wants to hear rather than their actual performance and behaviour in day to day practice. Besides KAP, barriers such as cultural and religious beliefs towards contraception may exist, which has not been addressed by this study. This is a call for further study to observe other variables such as culture and religion using the KAP approach.

### Recommendations

The outcomes of this study are presumed to aid in assessing the current level of KAP of the CPPs towards ECP that demands refinement. Educational campaigns focusing the pharmacists and other healthcare professionals are imperative to enhance the knowledge, improve the dispensing practice and exterminate the misbelief of the CPPs towards ECP, which will help to loosen the existing reservation notions. Furthermore, the curricula relating to reproductive medicines and contraceptives should be strong as finding from this study showed that the significant source of information of most of the respondents was textbooks.

## Conclusion

Emergency contraceptive pills (ECPs) are a widely used contraceptive measure in Nepal where the incidence of unwanted pregnancies in on the rise. Community pharmacies are an essential access point for obtaining ECP because of its’ flexible time of operation and its’ closer proximity than the other healthcare facilities, and they play a significant role in providing ECP and promoting its’ rational use. The findings conclude that knowledge is a crucial element to improve the dispensing practice of ECP. Majority of the respondents had good knowledge and good practice on dispensing ECP and possessed a positive attitude towards the use of ECP. However, some of the respondents felt that ECP should not be categorized under OTC drugs and agreed that ECP without prescription would promote unsafe sex and the CPPs lacked specific information about ECP such as side effects, dosing schedule, time frames. This could affect the information given during counselling by the CPPs to their users. A lack of knowledge can result in the delivery of misinformation to ECP users and can result in inappropriate use of the drug, thus leading to unwanted pregnancies. Hence, educational intervention and awareness programs should be designed to educate pharmacy personnel on emergency contraception methods that are essential for providing excellent sexual and reproductive health services to women.

## Supplementary information

**Additional file 1.**

## Data Availability

The datasets used and/or analyzed during the current study are available from the corresponding author on reasonable request.

## Abbreviations

CMA: Community Medicine Assistant
D Pharmacy: Diploma in Pharmacy
DDA: Department of Drug Administration
EC: Emergency contraception
ECPs: Emergency Contraceptive Pills
NDHS: Nepal Demographic and Health Survey
OR: Odds Ratio
AOR: Adjusted Odds Ratio
COR: Crude Odds Ratio
OTC: Over the Counter
SPSS: Statistical package for social sciences
STI: Sexually Transmitted Infections
WHO: World Health Organization

## References

[1] Santelli J, Rochat R, Hatfield-Timajchy K, Gilbert BC, Curtis K, Cabral R, Hirsch JS, Schieve L (2003). The measurement and meaning of unintended pregnancy. Perspect Sex Reprod Health.

[2] Centers for Disease Control. Unintended Pregnancy. [https://www.cdc.gov/reproductivehealth/contraception/unintendedpregnancy/index.htm]. Accessed 2 July 2020.

[3] Finer LB, Zolna MR (2011). Unintended pregnancy in the United States: incidence and disparities, 2006. Contraception.

[4] Klima CS (1998). Unintended pregnancy: consequences and solutions for a worldwide problem. J Nurse-Midwifery.

[5] Singh S, Sedgh G, Hussain R (2010). Unintended pregnancy: worldwide levels, trends, and outcomes. Stud Fam Plan.

[6] Nepal Demographic and Health Survey (NDHS). 2011. [https://dhsprogram.com/pubs/pdf/fr257/fr257%5B13april2012%5D.pdf]. Accessed 1 July 2020.

[7] Shrestha DR, Regmi SC, Dangal G (2018). Abortion: still unfinished agenda in Nepal. J Nepal Health Res Counc.

[8] Abortion and Unintended Pregnancy in Nepal. Center for Research on Environment Health and Population Activities 2017. [https://www.guttmacher.org/factsheet/abortion-unintended-pregnancy-in-nepal]. Accessed 1 July 2020.

[9] Center for Research on Environment, Health and Population Activities (CREHPA). National Workshop on developing sustainable strategies for introducing emergency contraception in Nepal. Workshop report, Kupondole 16 December. Lalitpur: CREHPA; 2004.

[10] Emergency contraception. [https://www.who.int/news-room/fact-sheets/detail/emergency-contraception]. Accessed 1 July 2020.

[11] World Health Organization. Emergency contraception: a guide for service delivery. In: World Health Organization; 1998. [https://apps.who.int/iris/handle/10665/64123]. Accessed 2 July 2020.

[12] Thapa S (2016). A new wave in the quiet revolution in contraceptive use in Nepal: the rise of emergency contraception. Reprod Health.

[13] Parker C. Adolescents and emergency contraceptive pills in developing countries. Family Health International; 2005.

[14] Rafie S, Stone RH, Wilkinson TA, Borgelt LM, El-Ibiary SY, Ragland D (2017). Role of the community pharmacist in emergency contraception counseling and delivery in the United States: current trends and future prospects. Integr Pharm Res Pract.

[15] Hobbs MK, Taft AJ, Amir LH, Stewart K, Shelley JM, Smith AM, Chapman CB, Hussainy SY (2011). Pharmacy access to the emergency contraceptive pill: a national survey of a random sample of Australian women. Contraception.

[16] Shrestha S, Dangol R, Shakya D, Danekhu K, Sharma S, Bhuvan K (2019). Bibliometric analysis of community pharmacy research activities in Nepal over a period of 1992-2018. J Karnali Acad Health Sci.

[17] Latthe M, Latthe P, Charlton R. Quality of information on emergency contraception on the Internet. Br J Fam Plann. 2000;26(1):39–43. . 10781966

[18] Omotoso O, Ajuwon AJ. Emergency contraceptive pill knowledge, attitudes and dispensing practices of pharmacists in Ibadan and Lagos metropolis, Nigeria. Sierra Leone J Biomed Res. 2010;2(2):135–41.

[19] Nepal Pharmacy Council. [http://nepalpharmacycouncil.org.np/]. Accessed 3 July 2020.

[20] Nepal Chemist and Druggist Association. [http://ncda.org.np/]. Accessed 8 July 2020.

[21] Gyawali S, Rathore DS, Bhuvan KC, Shankar PR. Study of status of safe injection practice and knowledge regarding injection safety among primary health care workers in Baglung district, western Nepal. BMC Int Health Hum Rights. 2013;13:3. 10.1186/1472-698X-13-3 PMC3583689 23286907

[22] Poudel A, Khanal S, Alam K, Palaian S. Perception of Nepalese community pharmacists towards patient counseling and continuing pharmacy education program: a multicentric study. J Clin Diagn Res. 2009;3(2):1408–13.

[23] Sakun S, Sandhya S, Nirsuba G. Knowledge Regarding Emergency Contraceptive among Women of Tanahu, Nepal; Pinnacle Medicine & Medical Sciences. 2014.

[24] Bhatta R, Godar S, Aryal K. Knowledge and practice regarding the use of emergency contraception among the higher secondary students of Nepal. Int J Comm Med Public Health. 2019;6(7):2751–4.

[25] Center for Research on Environment Health and Population Activities. [www.crehpa.org.np] Accessed 10 July 2020.

[26] Raosoft Sample Size Calculator. [http://www.raosoft.com/samplesize.html]. Accessed 11 Jan 2018.

[27] Ehrle N, Sarker M (2011). Emergency contraceptive pills: knowledge and attitudes of pharmacy personnel in Managua, Nicaragua. Int Perspect Sex Reprod Health.

[28] Taber KS (2018). The use of Cronbach’s alpha when developing and reporting research instruments in science education. Res Sci Educ.

[29] Belachew SA, Yimenu DK, Gebresillassie BM (2017). Pharmacy Professionals’ dispensing practice, knowledge, and attitude towards emergency contraceptives in Gondar town, Northwestern Ethiopia: A Cross-Sectional Study. Int J Reprod Med.

[30] Apikoglu-Rabus S, Clark PM, Izzettin FV (2012). Turkish pharmacists’ counseling practices and attitudes regarding emergency contraceptive pills. Int J Clin Pharm.

[31] Szűcs M, Párduczné Szöllősi A, Bártfai G (2010). Knowledge and attitudes of pharmacists regarding over-the-counter emergency contraception in south-eastern Hungary. Eur J Contracept Reprod Health Care.

[32] Kishore V, Misro MM, Nandan D. Providers’ knowledge, attitude and dispensing practices of E-pills in government dispensaries of south district in Delhi, India. Indian J Community Med. 2010;35(1):46–51. 10.4103/0970-0218.62553 PMC2888367 20606919

[33] Yam EA, Gordon-Strachan G, McIntyre G, Fletcher H, Garcia SG, Becker D, Ezcurra E. Jamaican and Barbadian health care providers’ knowledge, attitudes and practices regarding emergency contraceptive pills. Int Fam Plan Perspect. 2007;33(4):160–7. 10.1363/ifpp.33.160.07 18178540

[34] Borrego ME, Short J, House N, Gupchup G, Naik R, Cuellar D. New Mexico pharmacists’ knowledge, attitudes, and beliefs toward prescribing oral emergency contraception. J Am Pharm Assoc. 2006;46(1):33–43. 10.1331/154434506775268634 16529339

[35] Blanchard K, Harrison T, Sello M. Pharmacists’ knowledge and perceptions of emergency contraceptive pills in Soweto and the Johannesburg central Business District, South Africa. Int Fam Plan Perspect. 2005;31(4):172–8. 10.1363/3117205 16439344

[36] Hussainy SY, Stewart K, Chapman CB, Taft AJ, Amir LH, Hobbs MK, Shelley JM, Smith AM (2011). Provision of the emergency contraceptive pill without prescription: attitudes and practices of pharmacists in Australia. Contraception.

[37] Neupane IP (2018). Perceived health impacts of teenage pregnancy among married adolescents in Peri-urban areas of Kathmandu Valley. J Health Promotion.

[38] Hellerstedt WL, Van Riper KK. Emergency contraceptive pills: dispensing practices, knowledge and attitudes of South Dakota pharmacists. Perspect Sex Reprod Health. 2005;37(1):19–24. 10.1363/psrh.37.19.05 15888399

[39] Aneblom G, Lundborg CS, Carlsten A, Eurenius K, Tydén T (2004). Emergency contraceptive pills over-the-counter: practices and attitudes of pharmacy and nurse-midwife providers. Patient Educ Couns.

