# Supplementary material for: Knowledge, attitude and practice of emergency contraceptive pills among community pharmacy practitioners working in Kathmandu Valley: a cross-sectional study
Source: BMC Health Serv Res. 2020 Jul 29;20:699. doi: 10.1186/s12913-020-05543-5 (PMC7392703; doi:10.1186/s12913-020-05543-5)
Supplement: Supplementary file 1 — Additional file 1. [file 12913_2020_5543_MOESM1_ESM.docx]

# Appendix 1

# QUESTIONNAIRE

**Socio-demographic Characteristics**

1. Age: 🞏 <20 🞏 20-29 🞏 30-39 🞏 40-49 🞏 ≥50
2. Gender: 🞏 Male 🞏 Female
3. Religion: 🞏 Hindu 🞏 Buddhist 🞏 Muslim 🞏 Christian

🞏 Others (Please Specify): …………………..

1. Degree/ Education:

🞏 Bachelors of Pharmacy 🞏 CMA 🞏 Diploma of Pharmacy

🞏 Masters of Pharmacy 🞏 Others (Please Specify): ………………………….

1. Primary Position:

🞏 Staff 🞏 Manager/Supervisor

🞏 Owner 🞏 Others (Please Specify): …………………

1. Years of Experience: 🞏 < 5 years 🞏 5-10 years 🞏 >10 years
2. Location of pharmacy: 🞏 Inside city 🞏 Near Hospital 🞏 Periphery
3. District in which pharmacy is situated: 🞏 Kathmandu 🞏 Lalitpur 🞏 Bhaktapur

**Practice of Community Pharmacy Practitioners on Emergency Contraceptive Pills**

1. Have you ever dispensed Emergency Contraceptive Pills?

🞏 Yes 🞏 No

1. Which brand of ECP is sold the most?
   - I pill 🞏 E-72 🞏 ECON 🞏 Unwanted 72 🞏 Max-72 🞏 Feminor
   - Others (Please specify): …………………….
2. On average, how many ECPs do you dispense every day?

🞏 1 to 10 🞏 11 to 20 🞏 21 to 30 🞏 31 to 40 🞏 41 to 50 🞏 Above 50

1. Who are the most frequent clients?
   - Teenagers 🞏 Adult Women 🞏 Adult Men
2. Most often the products are sold on:

🞏 Patient request 🞏 Patient approaches with prescription

🞏 On your recommendations

1. Sources of information
   - Medical Representative (MR) 🞏 Text book 🞏 Training 🞏 Internet
2. Do you feel ECPs should be categorized under Over the Counter (OTC) drug?

🞏 Yes 🞏 No 🞏 Don’t Know

1. Received formal training/education on dispensing of ECP.
   - >1 year back 🞏 <1 year back 🞏 Not received
2. To whom you have provided ECPs: (Multiple answer possible)
   - Girls aged under 18 years
   - Men seeking ECPs for their partners
   - Women of any age requesting for ECP
   - Rape victims
   - Woman whose partner’s barrier contraception method fail
   - Women who did not use any contraception method
3. Do you think Counseling is important role of Pharmacist’s before dispensing ECPs?

🞏 Yes 🞏 No 🞏 Don’t Know

1. Do you counsel all ECPs users while dispensing?

🞏 Yes 🞏 No

1. Do you counsel on mechanism of action of ECPs?

🞏 Yes 🞏 No

1. Do you counsel the time at which the ECPs should be taken?

🞏 Yes 🞏 No

1. Do you counsel on side effects of ECPs?

🞏 Yes 🞏 No

**Knowledge of Community Pharmacy Practitioners on Emergency Contraceptive Pills**

1. Mechanism of action of ECP:

🞏 Prevent or Delay Ovulation (release of egg from an ovary)

🞏 Induce Abortion

🞏 Prevent an already established pregnancy

🞏 Don’t Know

1. How many times in the past years have you received information about ECP?

🞏 No 🞏 Yes, once 🞏 Yes, more than once

1. Do you know when must the pills be taken to be clinically effective?

🞏 Before unprotected sexual intercourse 🞏 During unprotected sexual intercourse

🞏 After unprotected sexual intercourse 🞏 Don’t Know

1. Within how many hours after unprotected sexual intercourse should the pills be taken?

🞏 5 🞏 24 🞏 48 🞏 72 🞏 120 🞏 Don’t Know

1. Mention the constituents of ECP.

🞏 Levonorgestrel 🞏 Levonorgestrel plus Ethinyl Estradiol 🞏 Don’t Know

1. What is the dose of ECPs?

🞏 Single dose of 1.5 mg or 2 doses of 0.75 mg

🞏 Single dose of 2.5 mg, or 2 doses of 1.5 mg

🞏 Don’t Know

1. ECP can harm a developing fetus :

🞏 Yes 🞏 No 🞏 Don’t Know

1. Situations where ECP can be used. ***(More than one answer possible)***

🞏 Missed Injection due date and had unprotected sex

🞏 Condom leaked/slipped

🞏 Rape victims

🞏 Intercourse without any family planning method

1. Do you know the side effects of ECPs?

🞏 Yes 🞏 No 🞏 Not Sure

If Yes: **(Please Specify any 2 side-effect if you know)**: ………………………………..

1. Does pill protect from Sexually Transmitted Infections (STI)?

🞏 Yes 🞏 No 🞏 Don’t Know

**Attitude of Community Pharmacy Practitioners on Emergency Contraceptive Pills**

1. ECPs are safe to use

🞏 Strongly Agree 🞏Agree 🞏 Neutral 🞏 Disagree 🞏 Strongly Disagree

1. Adolescents (Teenagers) should be given an easy access to ECPs

🞏 Strongly Agree 🞏Agree 🞏 Neutral 🞏 Disagree 🞏 Strongly Disagree

1. Adolescents (Teenagers) should be discouraged to use ECPS.

🞏 Strongly Agree 🞏Agree 🞏 Neutral 🞏 Disagree 🞏 Strongly Disagree

1. ECPs can be used as a regular oral routine contraceptive method (Nilocone white).

🞏 Strongly Agree 🞏 Agree 🞏 Neutral 🞏 Disagree 🞏 Strongly Disagree

1. Do you recommend ECPs use?

🞏 Strongly Agree 🞏 Agree 🞏 Neutral 🞏 Disagree 🞏 Strongly Disagree

1. Government of all countries should legalize ECPs?

🞏 Strongly Agree 🞏 Agree 🞏 Neutral 🞏 Disagree 🞏 Strongly Disagree

1. ECPs discourage regular contraceptive method use among youth.

🞏 Strongly Agree 🞏 Agree 🞏 Neutral 🞏 Disagree 🞏 Strongly Disagree

1. All sexually active women should be aware of ECP.

🞏 Strongly Agree 🞏 Agree 🞏 Neutral 🞏 Disagree 🞏 Strongly Disagree

1. Routine information about ECP should be included in contraceptive counseling.

🞏 Strongly Agree 🞏 Agree 🞏 Neutral 🞏 Disagree 🞏 Strongly Disagree

1. Information of ECP should be included in sex education in school.

🞏 Strongly Agree 🞏 Agree 🞏 Neutral 🞏 Disagree 🞏 Strongly Disagree

1. Are you uncomfortable dispensing ECP for moral or religious reason?

🞏 Strongly Agree 🞏 Agree 🞏 Neutral 🞏 Disagree 🞏 Strongly Disagree

1. Formal training is needed to enable the dispensers to appropriately dispense ECPs.

🞏 Strongly Agree 🞏 Agree 🞏 Neutral 🞏 Disagree 🞏 Strongly Disagree

1. ECP without prescription will promote unsafe sex.

🞏 Strongly Agree 🞏 Agree 🞏 Neutral 🞏 Disagree 🞏 Strongly Disagree

**Thank you for your time and patience!**
